# Adalimumab, Infliximab, and Vedolizumab in Treatment of Ulcerative Colitis: A Long-Term Retrospective Study in a Tertiary Referral Center

**DOI:** 10.1093/crocol/otab049

**Published:** 2021-07-13

**Authors:** Ann-Lorie Gagnon, William Beauchesne, Laurence Tessier, Charles David, Djamal Berbiche, Alexandre Lavoie, Alban Michaud-Herbst, Karine Tremblay

**Affiliations:** 1 Centre Intégré Universitaire de Santé et de Services Sociaux du Saguenay–Lac-Saint-Jean (Chicoutimi University Hospital), Research Centre, Saguenay, Quebec, Canada; 2 Parmacology–Physiology Department, Université de Sherbrooke, Saguenay, Quebec, Canada; 3 Centre de Recherche Charles-Le Moyne-Saguenay–Lac-Saint-Jean Sur Les Innovations en Santé (CR-CSIS), Université de Sherbrooke, Longueuil, Quebec, Canada; 4 Pharmacy Department, Centre Intégré Universitaire de Santé et de Services Sociaux du Saguenay–Lac-Saint-Jean (Chicoutimi University Hospital), Saguenay, Quebec, Canada; 5 Gastroenterology Department, Centre Intégré Universitaire de Santé et de Services Sociaux du Saguenay–Lac-Saint-Jean (Chicoutimi University Hospital), Saguenay, Quebec, Canada

**Keywords:** ulcerative colitis, adalimumab, infliximab, vedolizumab, population-based study

## Abstract

**Background:**

Biological therapies have changed the landscape of pharmacological management of ulcerative colitis (UC). However, a large proportion of patients do not respond to biologics, lose their response over time, or present adverse drug events. This study aims to assess therapeutic response and treatment persistence to adalimumab, infliximab, and vedolizumab, 3 agents widely used in a tertiary referral center of Saguenay–Lac-Saint-Jean (Quebec, Canada).

**Methods:**

We conducted a retrospective population-based study with a thorough review of patients’ medical charts. Adults at UC diagnosis, with current or past use of adalimumab, infliximab, or vedolizumab, were included in the study. Clinical data were collected in order to assess response phenotypes and persistence to treatment. Kaplan–Meier curves were performed to assess treatment persistence, and predictors for discontinuation were assessed using Cox regression analyses.

**Results:**

A total of 134 patients were included in this study. For the cases exposed to adalimumab, infliximab, and vedolizumab, 56.9%, 62.5%, and 47.5% were responders, respectively. Mean persistence rates (95% CI) were 5.5 (4.3–6.6), 10.1 (8.7–11.5), and 3.6 (2.9–4.2) years for adalimumab, infliximab, and vedolizumab, respectively. Increased persistence rates were observed in biologic-naïve patients treated with infliximab in comparison to those with the previous exposition to 2 biologics, but no such effect was observed for adalimumab or vedolizumab. Overall, 61.9% of cases had adverse drug events and of these, 6 led to treatment discontinuation.

**Conclusion:**

This study presents long-term treatment persistence data with adalimumab, infliximab, and vedolizumab, showing that more than half of cases treated with these biologics remained on treatment at least 24 months after initiation.

## Introduction

Ulcerative colitis (UC) is a chronic inflammatory disease characterized by inflammation of the mucosal layer of the colon and almost invariably involves the rectum and frequently extends to proximal segments of the colon.^1^ Management of UC varies according to severity and extent of the disease and aims to achieve both clinical and endoscopic remission.^2^ Patients with mild UC are typically treated with oral or topical 5-aminosalicylic acid, and topical or oral corticosteroids are added to patients who do not respond nor achieve remission on first-line treatment.^1^ For moderate to severe disease, corticosteroids can also be used to induce remission, but they are not adequate in the maintenance of remission, and thiopurines should be considered to maintain remission instead.^1^ Biological therapies, also known as biologics, such as monoclonal antibodies, have been shown to be effective in the induction and maintenance of remission in patients with UC, presenting suboptimal response or adverse drug reactions (ADRs) with conventional therapies.^1^ Biologics are now well established in the management of inflammatory bowel disease (IBD) and their use is associated with a reduction of IBD-related surgeries and hospitalizations.^3^

However, many patients do not respond favorably to these treatments. Among non-responders (NRs), 10%–40% present no improvement of their condition while 50%–80% do not achieve remission,^4^ representing a considerable number of patients. Two types of NR are generally recognized: (1) primary NR (or nonresponse) being patients in whom induction of remission is not achieved with therapy and (2) secondary NR (or loss of response [LOR]) being patients who, despite successful induction of remission, lose their response during the maintenance phase of therapy.^5^ Despite these different response phenotypes, medication persistence, defined as the time from drug initiation to discontinuation of therapy, can serve as a simple complementary parameter to assess real-world efficacy and safety profiles of therapies.^6^

In the Saguenay–Lac-Saint-Jean (SLSJ) region (region of Quebec Province, Canada), adalimumab, infliximab, and vedolizumab are chiefly used in the Gastroenterology Department of the “Centre intégré universitaire de santé et de services sociaux du Saguenay–Lac-Saint-Jean” (CIUSSS-SLSJ), a tertiary referral center, which provides care to every patient in the region with IBD. However, the extent to which biologics are used and their long-term efficacy have yet to be documented in this population. Therefore, this study aimed to assess therapeutic response and treatment persistence rates in a population-based cohort of patients treated with adalimumab, infliximab, and vedolizumab.

## Materials and Methods

### Study Design and Case Identification

We conducted a retrospective chart review population-based study in order to assess therapeutic response and treatment persistence to adalimumab, infliximab (including both REMICADE and INFLECTRA), and vedolizumab in patients with UC. The inclusion criteria were adults diagnosed with UC followed up in the gastroenterology service of the CIUSSS-SLSJ with past or present treatment with one of the 3 studied biologics. Subjects were identified from an institutional database, and a unique study denominalized identification number was attributed to each eligible case identified in order to protect confidentiality.

### Data Collection

Data were collected retrospectively from the opening of the patients’ medical file to September 30, 2020 and were captured into individualized paper case report forms (CRFs). Demographics, lifestyle habits, and comorbidities were collected during chart reviews. Assessment of alcohol consumption was made using Canada’s Low-Risk Alcohol Drinking Guidelines,^7^ and cases were divided into 4 categories of alcohol use: excessive (>15 drinks/week for men and >10 drinks/week for women), moderate (≤15 drinks/week for men and ≤10 drinks/week for women), former, and none. Tobacco and drug use were divided into 3 categories (current, former, and never) with no frequency distinction due to the scarcity of this information in medical charts. UC disease extent at diagnosis was reported following the Montreal classification.^8^ The use of corticosteroids (co-induction and concomitance) and immunosuppressant (co-induction) with biologics was reported when applicable. The use of golimumab, ustekinumab, and tofacitinib was considered in the amount of prior received treatments. All relevant clinical (outpatient clinics or gastroenterology consultations), endoscopic information (endoscopic report), and C-reactive protein (CRP) were collected in the CRF. CRP was collected at the date of treatment initiation when available or at the closest occurrence prior to treatment initiation (up to 3 months before initiation or else considered missing). Mayo scores were collected whenever available in patients’ medical charts or calculated with available relevant data.

### Response Phenotype Definitions

In the present study, a responder was defined by a Total Mayo score between 0 and 1 following at least 12 months of uninterrupted treatment with a single biologic agent. In cases without endoscopic reports in medical charts, those with a score of 0 on both the “stool frequency” and “rectal bleeding” parts of the Total Mayo score (eg, a normal number of daily stools and no rectal bleeding) were considered to be in clinical remission and thus, responders. A primary NR was defined by the failure to reach clinical or endoscopic remission (defined as Mayo Endoscopy subscore of 0 or 1) as well as manifesting no improvement during the induction period with the studied agent. A secondary NR was defined as cases with a LOR, that is, a Total Mayo score greater than 2 leading to agent discontinuation following successful induction of remission. However, in cases without endoscopic reports in medical charts, the loss of clinical remission (ie, an increase of daily stools or the reoccurrence of rectal bleeding) was considered as evidence of a LOR, and cases were accordingly classified as secondary NR. Cases with a Total Mayo score of 2, at least 12 months after initiation of medication, were assessed on a case-by-case basis. Lastly, cases that could not be categorized in the 3 response phenotypes described above were classified as intermediate phenotypes. These include cases that did not achieve clinical nor endoscopic remission but that displayed some degree of clinical or endoscopic improvement after 12 months of exposure. A sole observer reviewed phenotype classification of the cases.

Toxicity was reported when a temporal relationship between the administration of the studied biologics and the onset of the ADR was present. In addition, a clear indication of the potential toxic relationship in the medical chart was a discriminating information. Once reported in the CRF, ADRs were categorized into reaction type groups in order to protect case confidentiality and to avoid possible re-identification within small groups (*n* < 5).

### Persistence Rate Measurement

Treatment persistence was defined as the time from drug initiation (index date) to the earliest event among discontinuation (regardless of the documented reason for discontinuation), switching to another drug or at the end of data availability. Switching from one agent to another without interruption was considered as discontinuation of the initial agent, and the moment of therapy switch was considered as the index date of the succeeding therapy. Patients that were treated with a biologic agent but were lost to follow-up (eg, patient no longer followed up at the gastroenterology service of the CIUSSS-SLSJ) were considered as being on treatment up to the last moment of known treatment status.

### Statistical Analysis

Descriptive statistics were used to characterize the study UC patients’ sample. Means and standard deviations for continuous variables and frequencies with proportions for categorical variables were calculated. For the comparative analysis, Chi-Square and Mann–Whitney tests were performed depending on the nature of the data (categorical or continuous). The cumulative persistence rate was estimated using Kaplan–Meier analysis. Predictors of treatment persistence were assessed using multivariable Cox proportional-hazards regression. Statistical significance was fixed at *P* < .05 for all tests. The statistical analyses were performed using IBM SPSS Statistics version 25. A statistician (D.B.) has validated all statistics.

### Ethical Considerations

The study was approved by the Institutional Ethics Review Board of the CIUSSS-SLSJ.

## Results

### Case Characteristics

A total of 136 cases fulfilled the study’s inclusion criteria. Upon medical charts review, 2 cases were excluded due to medication noncompliance and thus, 134 cases were finally included in the study. Clinical and demographic characteristics are presented in Table 1. In brief, the mean age at diagnosis was 38 years old and more than half were men (59.0%; *n* = 79). About half of the cases had moderate alcohol consumption (52.0%; *n* = 66), half had never used tobacco products (52.4%; *n* = 66), and a vast majority had never used drugs on a regular basis (94.2%; *n* = 114). At the moment of diagnosis, pancolitis was present in 45.7% of cases while 38.8% presented with left-sided colitis and 15.5% had proctitis. Respiratory disease (20.9%; *n* = 28), including asthma and obstructive lung disease, and high blood pressure (20.1%; *n* = 27) were the most reported comorbidities. Diabetes, cardiovascular disease, arthritis, and cancer (including breast, prostate, lung, lymph node, liver, biliary tract, and bladder cancers) were noted in less than 10% of cases. Infliximab was the first biologic agent used in 44.0% of cases closely followed by adalimumab in 38.1%, while 14.9% used vedolizumab as their first biologic treatment. Cases treated with adalimumab and infliximab were mostly naïve to biologics or tofacitinib, with 82.8% and 79.4% of cases, respectively, as opposed to vedolizumab cases with only 56.4% with at least one prior biologic or tofacitinib exposure. A colectomy was performed in 7.5% of cases (*n* = 10) of which 4 were partial colectomies due to benign stenosis on diverticular disease, inflammatory stenosis and suspected dysplasia, previous history of colonic intestinal perforation, and cecal perforation on colonoscopy.

**Table 1.** Characteristics of ulcerative colitis patients treated with adalimumab, infliximab, and vedolizumab.

	UC patients (*n* = 134)
*Demographic parameters*	
Age at study inclusion, mean years (SD)	50.0 (17.1)
Age at UC diagnosis, mean years (SD)	38.2 (16.4)
Sex, *n* (%)	
Male	79 (59.0)
Female	55 (41.0)
*Life habits*	
Alcohol use, *n* (%)^a^	
Excessive	5 (3.9)
Moderate	66 (52.0)
Former	19 (15.0)
Never	37 (29.1)
Tobacco use, *n* (%)^a^	
Current	14 (11.1)
Former	46 (36.5)
Never	66(52.4)
Drugs use, *n* (%)^a^	
Current	4 (3.4)
Former	3 (2.5)
Never	114 (94.2)
*Medical parameters*	
Extent at diagnosis, *n* (%)^a^	
E1—Proctitis	18 (15.5)
E2—Left-sided colitis	45 (38.8)
E3—Pancolitis	53 (45.7)
Comorbidities, *n* (%)	
Respiratory disease	28 (20.9)
High blood pressure	27 (20.1)
Diabetes	13 (9.7)
Cardiovascular disease	13 (9.7)
Arthritis	12 (9.0)
Cancer	7 (4.6)
*Treatment exposure*	
First biological used, *n* (%)	
Adalimumab	51 (38.1)
Infliximab	59 (44.0)
Vedolizumab	20 (14.9)
Others^b^	4 (3.0)
Colectomy, *n* (%)	10 (7.5)

Abbreviations: SD, standard deviation; UC, ulcerative colitis.

Proportion calculated on total available data.

Includes exposure to golimumab.

### Efficacy Assessment

**Figure 1.**
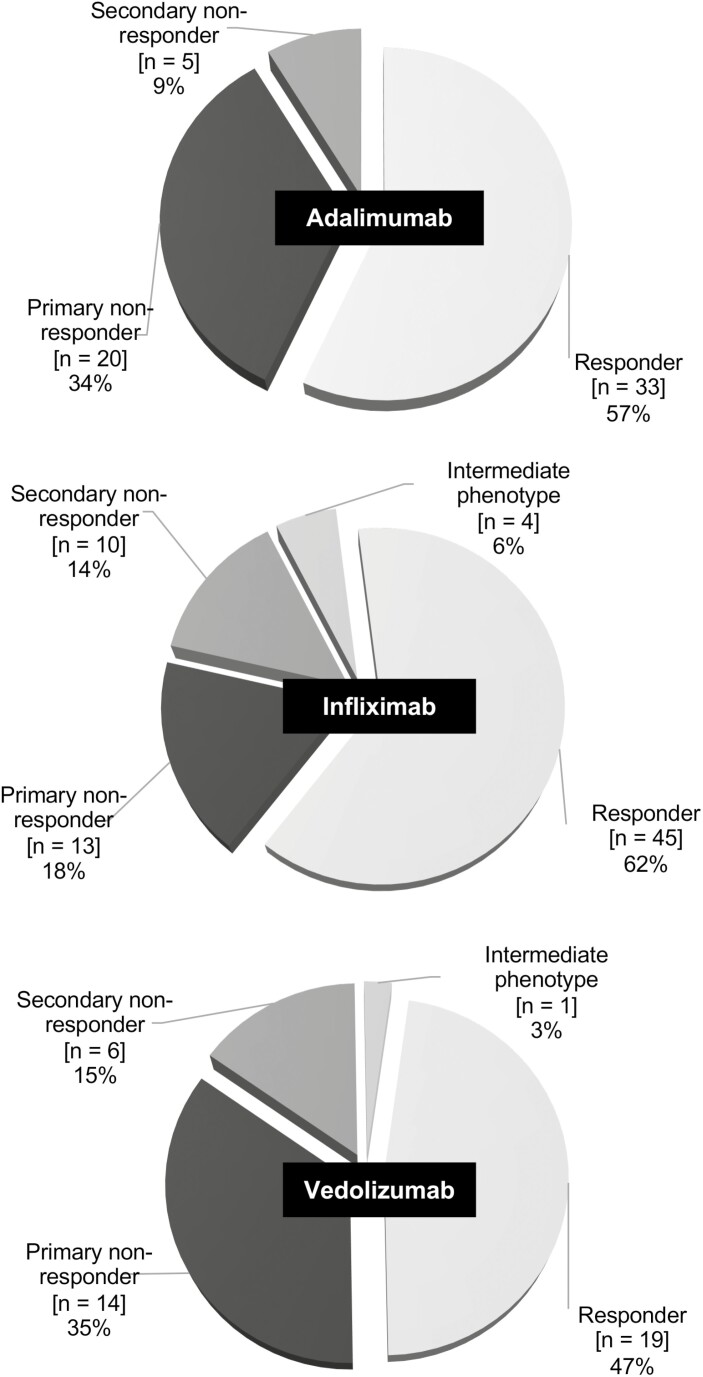
Efficacy phenotypes distribution among ulcerative colitis subjects exposed to studied biologics. Circular graphs show the proportion of each response phenotype observed for adalimumab, infliximab, and vedolizumab.

A decision tree has been developed to standardize the assessment of response phenotypes from data available in medical charts (Figure S1). A gastroenterologist validated the decision tree and response phenotypes of each subject were assessed using the tool. As shown in Figure 1, 33 (56.9%) cases exposed to adalimumab were responders, while 20 (34.5%) were primary NR and 5 (8.6%) secondary NR. Two adalimumab cases were classified as responders, despite having less than 12 months of exposure (10 and 11 months) based on clinical judgment. For infliximab, 45 (62.5%) cases were responders, 13 (18.1%) were primary NR, 10 (13.9%) secondary NR, and 4 (5.6%) had an intermediate phenotype. Finally, when exposed to vedolizumab, 19 (47.5%) cases were responders, 14 (35.0%) were primary NR, 6 (15.0%) secondary NR, and 1 (2.5%) had an intermediate phenotype. There were no statistically significant differences in the proportion of responders and NR between the 3 biologic groups (*P* = .205). One vedolizumab case was classified as a responder despite having had only 9 months of exposure based on clinical judgment. Median time to futility (ie, time before LOR leading to discontinuation of the agent used) was 3.9 years for adalimumab (*n* = 5; range 0.8–7.9 years), 2.4 years for infliximab (*n* = 10; range 0.8–12.0 years), and 1.3 years for vedolizumab (*n* = 6; range 0.5–1.9 years).

Relevant variables potentially associated with biologic response are presented in Table 2. Owing to the low prevalence of secondary NR, primary and secondary NR were combined into a single NR group, and cases with intermediate phenotype (*n* = 5) were not considered in these analyses. Corticosteroid co-induction with infliximab or vedolizumab was associated with nonresponse (87.0% vs 53.3%, *P* = .007 for infliximab; 73.7% vs 35.3%, *P* = .042 for vedolizumab). A greater proportion of NRs in the vedolizumab group had baseline CRP value greater than 5mg L^−1^ (85.7% vs 40.0%, *P* = .032), and CRP values were also significantly higher in NRs to vedolizumab (42.0 ± 40.5 vs 8.4 ± 8.9, *P* = .009, data not shown). However, baseline CRP value greater than 5mg L^−1^ was not associated with response phenotypes for infliximab or adalimumab nor were baseline CRP values higher in NR patients. Therapy regimen modification was not associated with response phenotypes nor was the presence of a hospitalization related to UC in the 3 months prior to therapy initiation. There was a greater proportion of infliximab cases with hospitalization due to UC in the 3 months prior to treatment initiation than adalimumab cases (22.9% vs 6.15%, *P* = .008, data not shown), and a greater proportion of infliximab cases presented a change to their treatment regimen in comparison to vedolizumab cases (83.3% vs 58.5%, *P* = .016, data not shown). No significant differences were observed between studied groups for the remaining variables.

**Table 2.** Comparative analyses of variables potentially associated with response phenotype to biologics.

	Adalimumab (*n* = 58)			Infliximab (*n* = 68)			Vedolizumab (*n* = 39)		
	R (*n* = 33)	NR (*n* = 25)	P	R (*n* = 45)	NR (*n* = 23)	P	R (*n* = 19)	NR (*n* = 20)	P
Age at UC diagnosis, mean years (SD)	40.7 (15.9)	35.5 (13.5)	.252	35.0 (14.7)	36.8 (16.0)	.636	36.8 (18.6)	39.9 (19.2)	.444
Age at treatment onset, mean years (SD)	47.3 (16.3)	43.9 (14.1)	.413	40.4 (14.7)	43.3 (15.2)	.454	45.8 (21.7)	45.5 (17.9)	.960
Sex, n (%)									
Male	20(60.6)	13 (52.0)	.597	30 (66.7)	11 (47.8)	.191	9 (47.4)	12 (60.0)	.527
Female	13 (39.4)	12 (48.0)		15 (33.3)	12 (52.2)		10 (52.6)	8 (40.0)	
Active tobacco use, *n* (%)^a^	4 (12.5)	1 (4.5)	.638	5 (11.4)	4 (18.2)	.467	2 (11.1)	4 (22.2)	.658
Active alcohol use, *n* (%)^a^	21 (65.6)	10 (43.5)	.168	22 (50.0)	11 (47.8)	1.000	11 (61.1)	8 (42.1)	.330
Extent at diagnosis, *n* (%)^a^									
E1—Proctitis	7 (25.0)	4 (16.7)	.285	4 (9.8)	4 (18.2)	.248	3 (21.4)	2 (11.1)	.175
E2—Left-sided colitis	9 (32.1)	13 (54.2)		18 (43.9)	5 (22.7)		7 (50.0)	5 (27.8)	
E3—Pancolitis	12 (42.9)	7 (29.2)		19 (46.3)	13 (59.1)		4 (28.6)	11 (61.1)	
Prior treatment, n (%)^b^									
No prior exposure	27 (81.8)	21 (84.0)	1.000	38 (84.4)	16 (69.6)	.160	9 (47.4)	8(40.0)	.909
One prior exposure	6 (18.2)	4 (16.0)		6 (13.3)	4 (17.4)		7 (36.8)	9 (45.0)	
Two prior exposure	0	0		1 (2.2)	3 (13.0)		3 (15.8)	3 (15.0)	
CRP >5mg mL^−1^ at baseline, *n* (%)^c^	9 (33.3)	8 (44.4)	.537	17 (58.6)	8 (47.1)	.545	4 (40.0)	12 (85.7)	**.032**
Dose optimization, *n* (%)	22 (66.7)	22 (88.0)	.116	38 (84.4)	18 (78.3)	1.000	8 (42.1)	15 (75.0)	.096
Co-induction with corticosteroids, *n* (%)	13 (39.4)	8 (33.3)	.782	15 (33.3)	11 (47.8)	.296	3 (18.8)	7 (36.8)	.285
Concomitance with corticosteroids, *n* (%)	20 (60.6)	13 (59.1)	1.000	24 (53.3)	20 (87.0)	**.007**	6 (35.3)	14 (73.7)	**.042**
Co-induction with Immunosuppressant, *n* (%)	4 (12.1)	2 (8.3)	1.000	13 (28.9)	5 (21.7)	.577	0	1 (5.3)	1.000
Hospitalization for UC, *n* (%)^c^	2 (6.1)	1 (4.0)	1.000	8 (17.8)	6 (26.1)	.527	3 (15.8)	1 (5.0)	.342

P-values under the specified threshold were typed using bold text.

Abbreviations: CRP, C-reactive protein; NR, non-responder; R, responder; SD, standard deviation; UC, ulcerative colitis.

Proportion calculated on total available data.

Includes exposure to golimumab, tofacitinib, ustekinumab, and the 3 studied molecules.

Within 3 months prior to agent initiation.

### Persistence Rate

**Figure 2.**
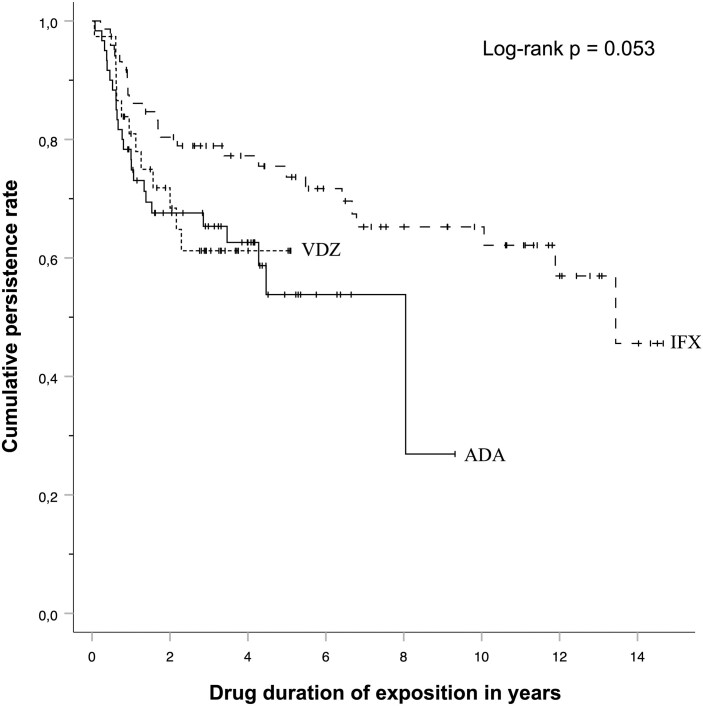
Kaplan–Meier curves of treatment persistence of studied biologics. Each curve presents the cumulative persistence rate according to drug exposure duration (in years) for adalimumab (ADA), infliximab (IFX), and vedolizumab (VDZ).

At the last moment of the chart review, 65.8% of cases exposed to infliximab remained on treatment while this proportion was 58.7% with adalimumab and 59.1% with vedolizumab. The persistence rate of each therapy is illustrated on the Kaplan–Meier plot (Figure 2). Mean persistence rates (95% CI) were 5.5 (4.3–6.6), 10.1 (8.7–11.5), and 3.6 (2.9–4.2) years for adalimumab, infliximab, and vedolizumab, respectively. The cumulative persistence rates were determined up until the fourth year after introduction of each agent. The cumulative proportions were 76.7%, 67.6%, 65.3%, and 62.6% at 1, 2, 3, and 4 years, respectively, after the initiation of adalimumab. The corresponding cumulative proportions were 86.1%, 80.4%, 78.9%, and 77.2% in the infliximab group and 81.0%, 71.8%, 61.2%, and 61.2% in the vedolizumab group. Comparison of persistence rates between the 3 studied agents shows a mild difference (*P* = .053).

Hazard ratios (HRs) of variables potentially associated with treatment discontinuation derived from a multivariable Cox regression are presented in Table 3. Left-sided colitis at diagnosis was associated with increased rates of discontinuation in comparison to pancolitis at diagnosis in patients treated with adalimumab (HR, 3.054; 95% CI, 1.096–8.505; *P* = .033), whereas the opposite was found for vedolizumab (HR, 0.100; 95% CI, 0.011–0.875; *P* = .038). For infliximab, prior exposure to 2 biologics, in comparison to cases with no prior exposure, was associated with increased rates of discontinuation (HR, 5.285; 95% CI, 1.103–25.324; *P* = .037). Age at onset was not associated with a significant change in the rates of discontinuation of any biologic agent group.

**Table 3.** Adjusted hazard ratios for treatment persistence according to the biologic agent used.

	Adalimumab		Infliximab		Vedolizumab	
	HR (95% CI)	P	HR (95% CI)	P	HR (95% CI)	P
Age at onset	0.974 (0.947–1.002)	.064	1.028 (0.997–1.060)	.073	1.006 (0.970–1.042)	.758
Extent at diagnosis^a^						
E1—Proctitis	1.440 (0.387–5.351)	.587	1.020 (0.322–3.231)	.973	0.360 (0.043–3.045)	.348
E2—Left-sided colitis	3.054 (1.096–8.505)	**.033**	0.337 (0.109–1.038)	.058	0.100 (0.011–0.875)	**.038**
E3—Pancolitis	Reference		Reference		Reference	
Prior treatment with NTA^b^						
No prior exposure	Reference		Reference		Reference	
One prior exposure	1.174 (0.382–3.610)	.779	2.449 (0.712–8.419)	.155	1.056 (0.267–4.180)	.938
Two prior exposure	–		5.285 (1.103–25.324)	**.037**	1.512 (0.263–8.704)	.644

P-values under the specified threshold were typed using bold text.

Abbreviations: HR = hazard ratio; NTA = new therapeutic avenues.

Proportion calculated on total available data.

Includes exposition to golimumab, tofacitinib, ustekinumab, and the 3 studied molecules.

### Adverse Drug Events

Overall, 83 cases (61.9%) had ADRs. The complete list of ADRs is given in Table 4. ADRs were most frequently reported in infliximab cases (63%; *n* = 45), followed by adalimumab (51%; *n* = 30) and vedolizumab (21%; *n* = 8). For infliximab, the most commonly reported ADRs were skin reactions (21.3%; *n* = 16) and musculoskeletal reactions (20.0%; *n* = 15) in addition to other reactions (24.0%; *n* = 18), such as asthenia. Neurological (20.4%; *n* = 11) and skin (16.7%; n = 9) reaction types were the most frequently reported ADRs in cases exposed to adalimumab. In vedolizumab cases, skin reactions (35.3%; *n* = 6) were the most frequently reported reaction type. ADRs were the cause of treatment discontinuation in 6 cases (4.5%), 4 of which linked to infliximab (16.0%), 1 to adalimumab (3.6%), and 1 to vedolizumab (6.3%).

**Table 4.** Adverse drug reactions types associated with adalimumab, infliximab, and vedolizumab.

	Adalimumab (*n* = 54)	Infliximab (*n* = 75)	Vedolizumab (*n* = 17)
Reaction type (*n* (%))^a^			
Skin^b^	9 (16.7)	16 (21.3)	6 (35.3)
Neurological^c^	11 (20.4)	12 (16.0)	2 (11.8)
Musculoskeletal^d^	5 (9.3)	15 (20.0)	3 (17.6)
Gastrointestinal^e^	7 (13.0)	4 (5.3)	2 (11.8)
Cardiovascular^f^	3 (5.6)	4 (5.3)	–
Site injection^g^	6 (11.1)	–	–
Infections^h^	1 (1.9)	3 (4.0)	–
Respiratory^i^	1 (1.9)	3 (4.0)	–
Other^j^	11 (20.4)	18 (24.0)	4 (23.5)

Proportion calculated on total available data.

Includes urticarial, dermatitis, psoriasis, acne, eczema, erythema, pruritus, dryness, and brown spots.

Includes vertigo, dizziness, headaches, confusion, hypoesthesia, dysarthria, memory loss, and absences.

Includes arthralgia and myalgia.

Includes nausea, vomiting, anorexia, gastroesophageal reflux, and dyspepsia.

Includes cardiopathy, peripheral edema, increased blood pressure, and palpitations.

Includes redness, swelling, itching, heat, and pain at site injection.

Includes cellulitis and recurrent infections.

Includes exacerbated asthma and dyspnea.

Includes asthenia, alopecia, gum edema, night sweats, hot flashes, shivers, dysphagia, and weight loss.

## Discussion

Biological therapies have drastically changed the landscape of pharmacological management of IBDs, especially for individuals with moderate to severe disease and those resistant to conventional therapies. In UC, adalimumab, infliximab, and vedolizumab have all been found to be effective in inducing and maintaining remission.^9^ However, there remains a significant proportion of patients that cannot be prospectively identified who do not respond to these biological therapies leading to unnecessary delay in adequate disease control. Significant heterogeneity in previous literature of long-term biologic use in UC regarding methodology, patient populations, data sources (database vs chart reviews), biologics studied, response phenotype definitions, and lengths of follow-up limited extensive comparison of our results. With an average follow-up period of -12 years, the present study reports the first long-term data regarding response phenotypes of patients using adalimumab, infliximab, or vedolizumab and their associated persistence rates in a well-characterized retrospective UC cohort from the SLSJ population (277 388 inhabitants in 2018,^10^ province of Quebec, Canada) since the CIUSSS-SLSJ Gastroenterology Department deserves the entire population.

A vast majority of adalimumab (82.8%) and infliximab (79.4%) users were naïve to biologics or tofacitinib at induction in contrast to vedolizumab users in whom slightly more than half (56.4%) had prior exposure to at least one agent. This observation is consistent with the recommendation of vedolizumab in patients with primary failure to an anti-tumor necrosis factor (TNF) therapy in Canadian guidelines.^11^ Also, the later federal regulatory approval and delayed coverage under the provincial public drug plan for vedolizumab, which happened after the anti-TNF of interest in this study, could have contributed to its lower frequency of use as a first-line therapy.

In our study, more than half of cases (56.9%) exposed to adalimumab were responders, which is similar to the few real-world studies published, where this proportion varies from 30% to 65% after 52 weeks of exposure.^12–18^ Similar results were observed with infliximab cases as more than half (62.5%) were also responders comparable to previous literature regarding the efficacy of infliximab in real-world studies reporting from 39% to 70% of responders.^19–24^ Lastly, the proportion of responders to vedolizumab (47.5%) in our cohort is also similar to most real-world studies, albeit the proportion of clinical remission after 52 weeks of treatment varies substantially from 9% to 67%.^25–36^ In a recent systematic review and network meta-analysis of first- and second-line therapies of moderate to severe UC, infliximab, adalimumab, and vedolizumab were all reported to be equally effective in the maintenance of remission in patients who responded to induction therapy,^9^ which is consistent with the absence of statistically significant differences in the proportions of responders between agents in this study. The greater proportion of NR among infliximab and vedolizumab cases with concomitant corticosteroids treatment could indirectly reflect the lower response rates observed with biologic use in patients with greater disease severity.^37^ However, no such association was observed in adalimumab cases. Nonresponse in vedolizumab users was associated with a baseline CRP value above 5mg L^−1^ along with higher CRP values, and this is in accordance with previous literature reporting that elevated CRP values at initiation of vedolizumab are generally negative predictors of clinical response.^38^ Despite the presence of a greater number of cases with hospitalization due to UC in the infliximab group in comparison to adalimumab, which could relate to increased disease severity in the infliximab cases, there was no significant difference in the proportion of NR between infliximab and adalimumab. This difference in the proportion of cases with hospitalization between the 2 anti-TNF could be explained by the tendency to use infliximab in hospitalized patients with UC, when the initiation of a biologic therapy is warranted.

Treatment persistence offers an indirect approach to assess long-term therapeutic benefits as a surrogate measure of drug efficacy in the real-world setting.^39^ A systematic review of real-world evidence on UC treatment persistence with biologics in the United States reported persistence rates at 1 year of therapy of 48%–84%,^40^ which is consistent with the 81.6% overall persistence rate at 1 year observed in the present study. Nevertheless, this is higher than what has been reported by Chen et al^41^ in a large claims database study comparing persistence rates between biologics, in which less than half of the patients continued biologic therapy after 1 year of treatment. Baer et al,^42^ using the Canadian longitudinal prescription claims database, reported a 69% and 33% persistence rate for infliximab at 1 and 5 years, respectively, both of which are inferior to those we observed in this study with infliximab (86.1% and 73.6%). Surprisingly, the lowest persistence rate we observed was for vedolizumab. This is opposed to previous literature evaluating real-world outcomes with biologics, whereas higher persistence rates were reported with vedolizumab in comparison to infliximab.^39, 41^ Some interesting relationships emerged when 3 potential determinants of long-term treatment persistence were examined in a multivariable regression analysis. Favorable treatment persistence was observed in biologic-naïve in comparison to patients treated with infliximab with 2 prior biologic treatments, but no such effect was found for adalimumab or vedolizumab. Previous anti-TNF therapy has been reported as a risk factor for treatment failure with anti-TNF agents,^37^ and the higher prevalence of NR cases using adalimumab as a first-line agent in comparison to infliximab (84.0% vs 69.6%) could have contributed to the divergence observed between the 2 anti-TNF agents. We report increased rates of treatment discontinuation in adalimumab cases with left-sided colitis at diagnosis compared to those with pancolitis and this was in opposition to vedolizumab cases, which had decreased rates of treatment discontinuation in the presence of left-sided colitis at diagnosis. While there has been a considerable interest in the potential link between disease extent and biologic treatment response, most studies found no association with some reporting opposite conclusions, ultimately making disease extent unreliable as a predictor of response at the moment.^37^ Finally, ADEs were the reason for agent discontinuation in only 6 cases, reinforcing the use of treatment persistence as an adequate proxy of drug efficacy in the present study.

There are some limitations to this study. Firstly, the sample sizes of individual biologics were limited and probably contributed to the discrepancies observed between therapies. This was unavoidable considering our convenience sampling of a subpopulation of patients with a disease of relatively low prevalence (322 per 100 000 inhabitants in Canada in 2018^43^). In fact, our sample (*n* = 134) is comparable to the estimated number of patients with UC treated with biologics based on the prevalence of biologic treatment among UC patients of 16.2% in the United States^44^ (expected 144 UC patients on biologics in the SLSJ population). Finally, the present study was observational in nature and carries the inherent biases associated with the retrospective study design. Objective markers of disease severity (eg, mucosal healing) were not readily available in medical charts and limited our investigations of the associations between disease activity and treatment response. This was mostly observed for the endoscopic assessment component of the Mayo score, as systematic recording of patient’s endoscopic subscores at the initiation of biologic treatment was implemented in 2015 in an effort to standardize clinical practice following the STRIDE initiative.^45^ Having said that, our study nevertheless provides insightful information on long-term persistence on 3 widely used biologic therapies. As such, considering the paucity of long-term studies of biologics in UC, the average follow-up period of 12 years of this study offers much needed long-term data while also being the first to report response phenotypes for adalimumab, infliximab, and vedolizumab and their associated persistence rates in a retrospective UC cohort from the SLSJ population (Quebec, Canada).

## Conclusions

We present the first population-based cohort with longitudinal data in Canada studying 3 of the most used biologics in UC, including data on biologic response phenotypes and treatment persistence. Overall, long-term treatment persistence with adalimumab, infliximab, and vedolizumab in this study was favorable with more than half of cases treated with these biologics remaining on treatment at least 24 months after their initiation. Large-scale prospective studies are necessary to assess long-term outcomes and thoroughly investigate predictors of durable treatment response.

## Supplementary Material

otab049_suppl_Supplementary_Materials

## Data Availability

The statistical data file, which contains clinical data collected from the medical chart review, can be made available by the corresponding author upon request. However, the archived medical charts of each case included in this study cannot be made available for consultation by others not being part of the research team in order to protect confidentiality.
