# Supplementary material for: Adalimumab, Infliximab, and Vedolizumab in Treatment of Ulcerative Colitis: A Long-Term Retrospective Study in a Tertiary Referral Center
Source: Crohns Colitis 360. 2021 Jul 13;3(4):otab049. doi: 10.1093/crocol/otab049 (PMC9802068; doi:10.1093/crocol/otab049)
Supplement: otab049_suppl_Supplementary_Materials [file otab049_suppl_supplementary_materials.docx]

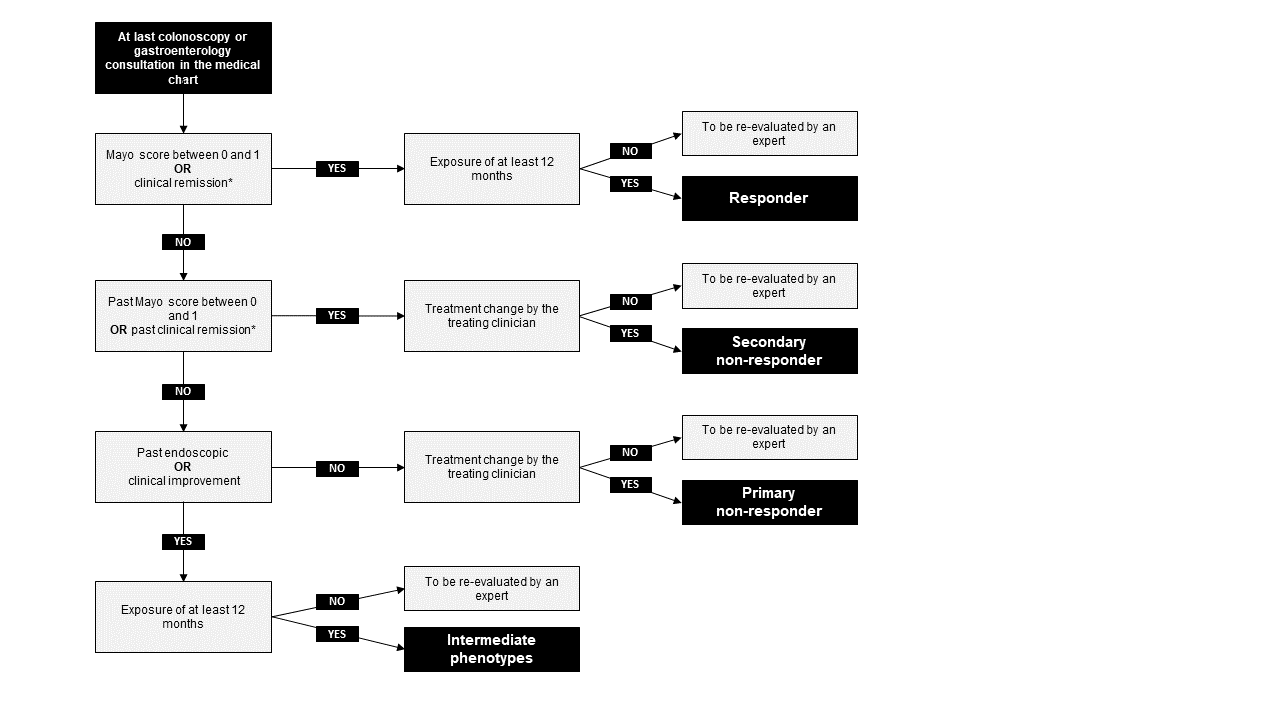


**Figure S1. Decision-making process tree used to assess the studied drugs’ response phenotypes**

*In cases without endoscopic reports in medical charts, those with a score of 0 on both the “Stool frequency” and “rectal bleeding” parts of the Mayo Score (e.g. normal number of daily stools and no rectal bleeding) were considered to be in clinical remission.
